# Characterization of competitive interactions in the coexistence of Bt-transgenic and conventional rice

**DOI:** 10.1186/s12896-015-0141-0

**Published:** 2015-04-26

**Authors:** Yongbo Liu, Feng Ge, Yuyong Liang, Gang Wu, Junsheng Li

**Affiliations:** State Key Laboratory of Environmental Criteria and Risk Assessment, Chinese Research Academy of Environmental Sciences, Beijing, 100012 China; State Key Laboratory of Integrated Management of Pest and Rodents, Institute of Zoology, the Chinese Academy of Sciences, 8 Dayangfang, Beijing, 100101 China; Institute of Plant Protection, Jiangxi Academy of Agricultural Sciences, Nanchang, 330200 China; Hubei Insect Resources Utilization and Sustainable Pest Management Key Laboratory, College of Plant Science and Technology, Huazhong Agricultural University, Wuhan, 430070 China

**Keywords:** Coexistence, Transgenic rice, Competitive ability, Herbivory, Agricultural ecosystem, Natural ecosystem

## Abstract

**Background:**

Transgene flow through pollen and seeds leads to transgenic volunteers and feral populations in the nature, and consumer choice and economic incentives determine whether transgenic crops will be cultivated in the field. Transgenic and non-transgenic plants are likely to coexist in the field and natural habitats, but their competitive interactions are not well understood.

**Methods:**

Field experiments were conducted in an agricultural ecosystem with insecticide spraying and a natural ecosystem, using *Bt*-transgenic rice (*Oryza sativa*) and its non-transgenic counterpart in pure and mixed stands with a replacement series.

**Results:**

Insect damage and competition significantly decreased plant growth and reproduction under the coexistence of transgenic and conventional rice. Insect-resistant transgenic rice was not competitively superior to its counterpart under different densities in both agricultural and natural ecosystems, irrespective of insect infection. Fitness cost due to *Bt*-transgene expression occurred only in an agroecosystem, where the population yield decreased with increasing percentage of transgenic rice. The population yield fluctuated in a natural ecosystem, with slight differences among pure and mixed stands under plant competition or insect pressure. The presence of *Chilo suppressalis* infection increased the number of non-target insects.

**Conclusions:**

Plant growth and reproduction patterns, relative competition ability and population yield indicate that *Bt*-transgenic and non-transgenic rice can coexist in agroecosystems, whereas in more natural habitats, transgenic rice is likely to outcompete non-transgenic rice.

## Background

As genetically modified (GM) crops have been cultivated worldwide, concerns over ecological consequences emerge because transgenic crops can establish a feral populations in the natural habitat [1-5], which might lead to coexistence with or even extinction of local species. The adventitious presence of GM material in non-GM crops is also a concern due to its economic implications. Without regulations, farmers can grow either GM or GM-free plants in their fields. The coexistence of GM and conventional crops depends on choices by farmers, which is influenced by consumer choice and economic incentives [6,7]. Proper isolation distance or pollen barriers between GM and non-GM fields should be ensured to minimize cross-fertilization so that the adventitious presence of transgenic materials in conventional products will be kept below the legal tolerance threshold, e.g. 0.9% in European Union (Commission Recommendation, 2003). To prevent insects from developing resistance to transgenic crops, the refuge strategy, where transgenic and non-transgenic crops coexist with each other, has usually been adopted [8,9]. Thus, transgenes might exist in landraces and wild relatives through gene flow and introgression by pollen dispersal [1-5,10] or seed movement [11,12]. Therefore, transgenic plants might coexist with non-transgenic plants in and out of the fields [3,7,13-16].

Transgenic crops are usually resistant to pests, herbicides, or diseases, which might enhance their fitness and promote the spread of transgenes into natural populations [17]. In mixture stands, however, the persistence of transgenic plants in a population depends on their relative fitness and competitive ability against non-transgenic neighbors. In general, insect-resistant plants have a competitive advantage and enhanced fecundity, particularly under high insect pressure [18-22]. Commercialization of *Bt*-transgenic crops, however, could reduce the abundance of target insects in agroecosystem [23,24]. This might influence the plant and insect relationship and the frequency of insect-resistant transgenic plants in mixed populations, which determines ecological risks and monitoring strategies [2].

Previous studies have been mainly focused on gene flow from transgenic crops to landraces and wild relatives and the coexistence of transgenic and non-transgenic progeny [1,3,20,22]. The long-term coexistence of transgenic and non-transgenic crops, however, has been largely ignored [15,25,26]. Moreover, ecological risk of crop-crop gene flow might be higher than that of crop-wild gene flow because no cross barrier exists between intraspecies crops. For example, pollen flow between transgenic and non-transgenic oilseed rapes (*Brassica napus*) results in the resistance of certain plants to three herbicides in the field [15].

GM rice (*Oryza sativa*) conferring insect-resistance, herbicide-tolerance, and high grain quality has been extensively developed in China [27], and two insect-resistant rice lines with *Bt* (*Bacillus thuringiensis*) transgenes were granted biosafety certificates in 2009. Thus, GM and non-GM rice are likely to coexist after the commercialization of GM crops, which might become a major concern over biosafety. This study aims to test (1) the relative competitive ability of insect-resistant *Bt*-transgenic to non-transgenic rice with different proportions of the former in mixed stands, (2) whether competition and herbivory pressures affect relative performance of *Bt*-transgenic to non-transgenic rice in pure and mixture stands, (3) whether increased frequency of transgenic plants in a population alters population productivity. The experiments were carried out in two settings: an agricultural ecosystem with insecticide spraying under local farming practice and a simulated natural ecosystem without insecticide use or other human disturbances.

## Results

### Plant growth and reproduction of transgenic and conventional rice

**Figure 1:**
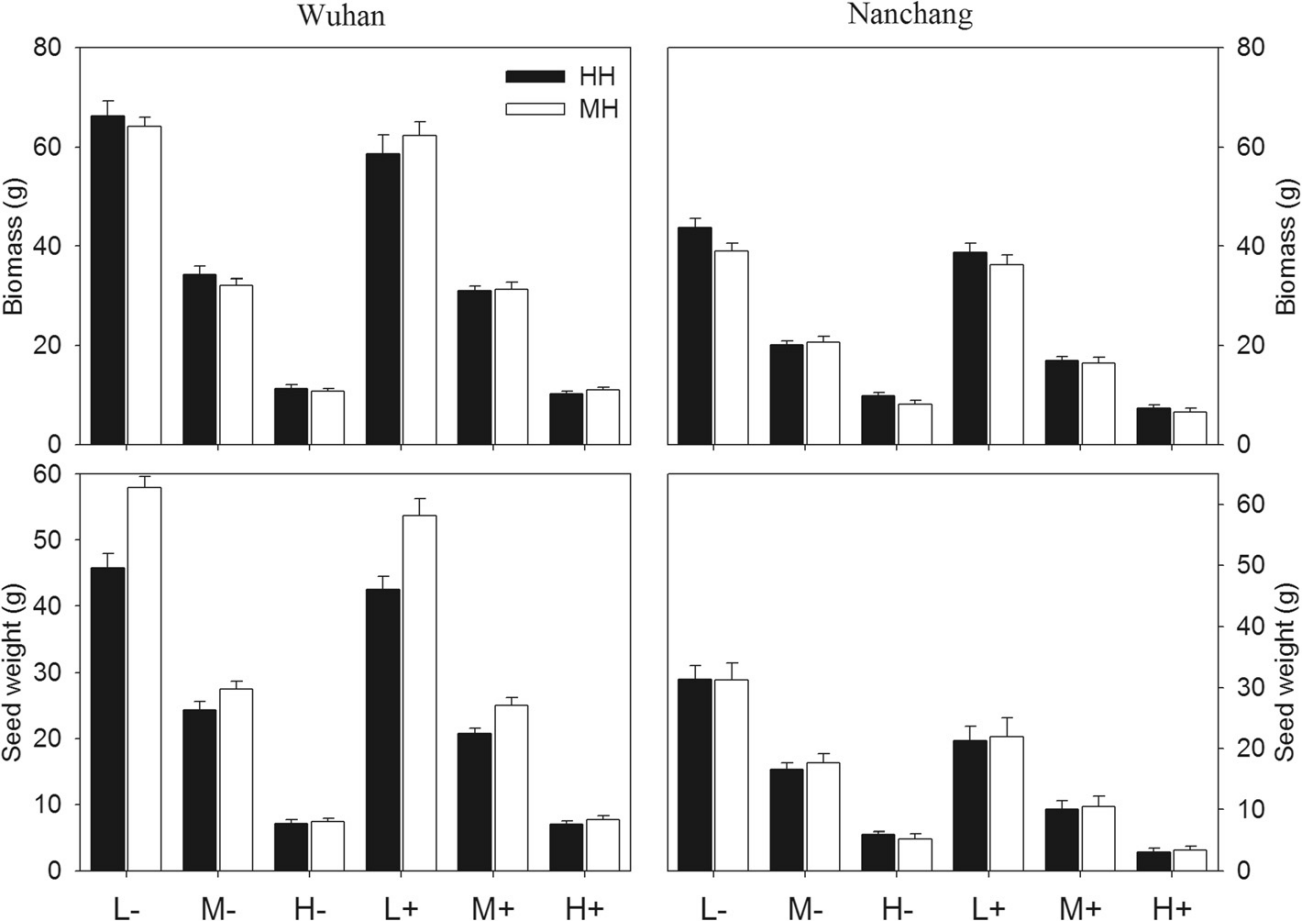
Biomass and seed weight of rice plants in Wuhan and Nanchang sites. Black bars, HH plants; white bars, MH plants. L, M, and H indicate low, medium and high densities respectively. “+” and “-” indicate the presence and absence of infection. Vertical bars denote SEM. See Table 2 for statistical significance.

Overall, rice plants performed better in the agriculturally managed site, Wuhan, than that in the natural site, Nanchang. Plants in Wuhan site grew higher and produced 20% more valid tillers, 60% more biomass, 50% more viable seeds and 80% more seed weight per plant on average and had a lower rate of invalid tillers and hollow seeds than that in Nanchang (Figure 1). Thus, subsequent analysis was conducted for Wuhan and Nanchang sites respectively.

**Figure 2:**
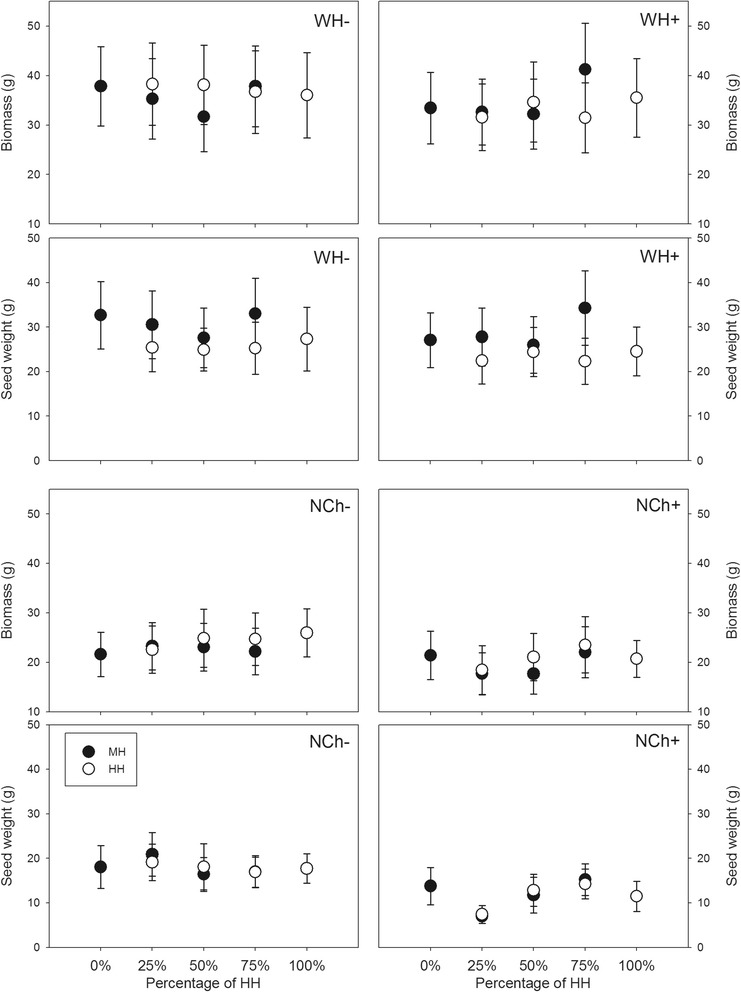
Biomass and seed weight in terms of the percentage of HH plants. Black points, MH plants; white points, HH plants. NCh, Nanchang; WH, Wuhan. “+” and “-” indicate the presence and absence of infection. Vertical bars denote SEM. See Table 1 and 2 for statistical significance.

Herbivory damage and plant competition significantly decreased plant growth and reproduction. In Wuhan, infection decreased plant height and seed weight and increased the rate of invalid tillers (RIT), but it had little effect on other characteristics (Table 1, Figure 1). Plant height, tiller number, biomass and ear length were not significantly different among five percentages of *Bt*-transgenic rice (*Oryza sativa*) Huahui-1 (HH), but the number of hollow seeds increased and seed weight and reproductive allocation (RA) decreased with increasing HH percentage (Table 1, Figure 2). The fitness parameters in high density plots, except for RIT, were significantly lower than that of plants in low density plots (Table 1). There was a significant interaction between infection and density for RIT and seed weight (Table 1). Insect pressure and plant competition had effects on plant growth and reproduction (Figure 1). Compared to non-transgenic Minghui-63 (MH), transgenic rice HH had more valid tillers and hollow seeds, lower RIT, less viable seeds and smaller seed weight (Table 1). There was significant interaction between plant type and infection for predicting RA; between HH percentage and plant type for predicting biomass and seed weight; between plant type and density for predicting RIT, seed weight and RA, respectively (Table 1).

**Table 1:** **Effect estimates and degrees of freedom of split-plot ANOVA for plant vegetation and reproductive growth in Wuhan site**

	**DF**	**RIT** ^**a**^	**Biomass**	**Ear length**	**Viable seeds**	**Hollow seeds**	**Seed weight**	**RA** ^**a**^
Infection (I)	1	41.9^*^	3.60	3.59	2.28	0.44	24.6^*^	0.05
Percentage(P)	4	1.19	0.97	0.34	1.50	5.26^**^	4.40^*^	3.48*
I × P	4	0.73	0.65	0.10	0.92	0.85	1.14	0.21
Density (D)	2	51.9^***^	1219^***^	92.7^***^	34.4^***^	42.2^***^	1710^***^	22.5^***^
I × D	2	4.44^*^	2.18	1.49	0.75	0.24	3.90^*^	2.72
P × D	8	0.58	1.05	1.28	0.54	1.83	2.23^*^	1.80
I × P × D	8	0.46	0.56	0.52	0.68	0.37	2.03	1.36
Plant type (T)	1	24.6^***^	0.00	0.30	19.9^***^	64.4^***^	29.8^***^	107^***^
I × T	1	0.04	3.10	0.01	0.01	0.16	0.27	5.88*
P × T	2	0.31	3.38^*^	0.87	1.23	0.20	4.54^*^	0.59
D × T	2	3.63^*^	0.30	0.15	2.31	1.97	14.7^***^	11.8^***^
I × P × T	2	0.47	0.24	0.03	0.06	0.03	0.56	0.04
I × D × T	2	3.07	0.35	1.83	1.12	0.25	0.24	0.66
P × D × T	4	2.46	0.53	1.31	0.38	0.16	0.59	0.61
I × P × D × T	4	1.12	0.47	0.81	0.99	1.39	0.76	0.79

^*^, ^**^and ^***^indicate significant difference at the P < 0.05, P < 0.01 and P < 0.001 levels.

^a^RIT, indicates the rate of invalid tillers; RA, reproductive allocation.

The increased frequency of transgenic rice had little effect on rice growth and reproduction. In the Nanchang site, as the percentage of transgenic plants increased, plants did not show significant difference in any variable (Table 2, Figure 2). Infection increased RIT and the number of hollow seeds and decreased seed weight and RA, but had little effect on other characteristics (Table 2, Figure 1). The plant fitness parameters in high density plots, except for RIT, were significantly lower than that of the plants in low density plots (Table 2). There was significant interaction between infection and density for RIT and seed weight. Insect pressure and plant competition had additive effects on plant growth and reproduction (Figure 1). There was little difference in the performance of HH and MH in the natural ecosystem (Table 2). Little interaction occurred among infection, percentage, density and plant type, except between percentage of transgenic plants and plant type for RIT and hollow seeds, and between density and plant type for biomass (Table 2).

**Table 2:** **Effect estimates and degrees of freedom of split-plot ANOVA for plant vegetation and reproductive growth in Nanchang site**

	**DF**	**RIT** ^**a**^	**Biomass**	**Ear length**	**Viable seeds**	**Hollow seeds**	**Seed weight**	**RA** ^**a**^
Infection (I)	1	30.1^*^	4.44	16.7	2.54	75.6^*^	26.0^*^	148^**^
Percentage(P)	4	1.35	1.31	2.17	0.61	1.86	0.33	0.62
I × P	4	1.63	1.25	1.28	1.43	0.96	1.64	1.60
Density (D)	2	317^***^	873^***^	55.1^***^	28.6^***^	17.4^***^	239^***^	23.8^***^
I × D	2	17.2^***^	0.91	1.70	0.31	0.01	6.78^**^	0.99
P × D	8	1.70	1.12	1.90	1.75	0.61	1.11	0.91
I × P × D	8	2.68^*^	1.44	2.23^*^	0.63	1.96	1.35	0.90
Plant type (T)	1	0.19	3.46	0.01	2.25	3.05	0.01	0.43
I × T	1	0.00	0.17	1.97	3.59	1.30	0.03	0.05
P × T	2	5.56^**^	0.89	2.72	2.88	4.38^*^	0.78	1.19
D × T	2	1.08	3.32^*^	0.08	1.07	1.34	0.08	1.30
I × P × T	2	0.33	0.27	2.27	0.36	4.21^*^	0.44	0.10
I × D × T	2	0.06	0.05	1.55	1.09	1.74	0.20	0.72
P × D × T	4	0.92	0.46	0.46	0.43	0.19	0.96	0.09
I × P × D × T	4	1.32	0.31	0.27	0.43	0.52	1.39	1.22

^*^, ^**^and ^***^indicate significant difference at the P < 0.05, P < 0.01 and P < 0.001 levels.

^a^RIT, indicates the rate of invalid tillers; RA, reproductive allocation.

### Relative competitive ability of insect-resistant rice to conventional rice

There was little difference in competitive ability between *Bt*-transgenic and conventional rice. The relative difference (RD) between HH and MH did not significantly differ from 0 (P > 0.05, *t*-test) under three densities (0.30 m, 0.20 m and 0.10 m space) in the two insect experiments of Wuhan and Nanchang sites. The (relative competitive ability) RCC values did not significantly deviate from 1 (P > 0.05, *t*-test) under three densities for biomass and seed production, with or without insects (Table 3), suggesting that HH rice was not competitively superior to MH plants in the mixed stands.

**Table 3:** **Mean (±SD) of relative crowding coefficient (RCC) between Bt-transgenic and conventional rice**

**Infection**	**Density**	**Wuhan**		**Nanchang**	
		**Biomass**	**Seed weight**	**Biomass**	**Seed weight**
No	Low	1.11 ± 0.05	1.33 ± 0.34	0.96 ± 0.24	1.10 ± 0.67
	Medium	1.02 ± 0.06	0.94 ± 0.23	0.94 ± 0.26	1.03 ± 0.50
	High	1.04 ± 0.09	1.08 ± 0.45	0.67 ± 0.24	1.14 ± 0.87
Yes	Low	1.02 ± 0.04	1.15 ± 0.49	1.39 ± 0.42	1.25 ± 0.40
	Medium	0.98 ± 0.03	0.89 ± 0.21	0.94 ± 0.32	1.43 ± 0.41
	High	1.02 ± 0.06	0.87 ± 0.28	0.94 ± 0.51	1.34 ± 0.66

### Plot production under the coexistence of transgenic and conventional rice

**Figure 3:**
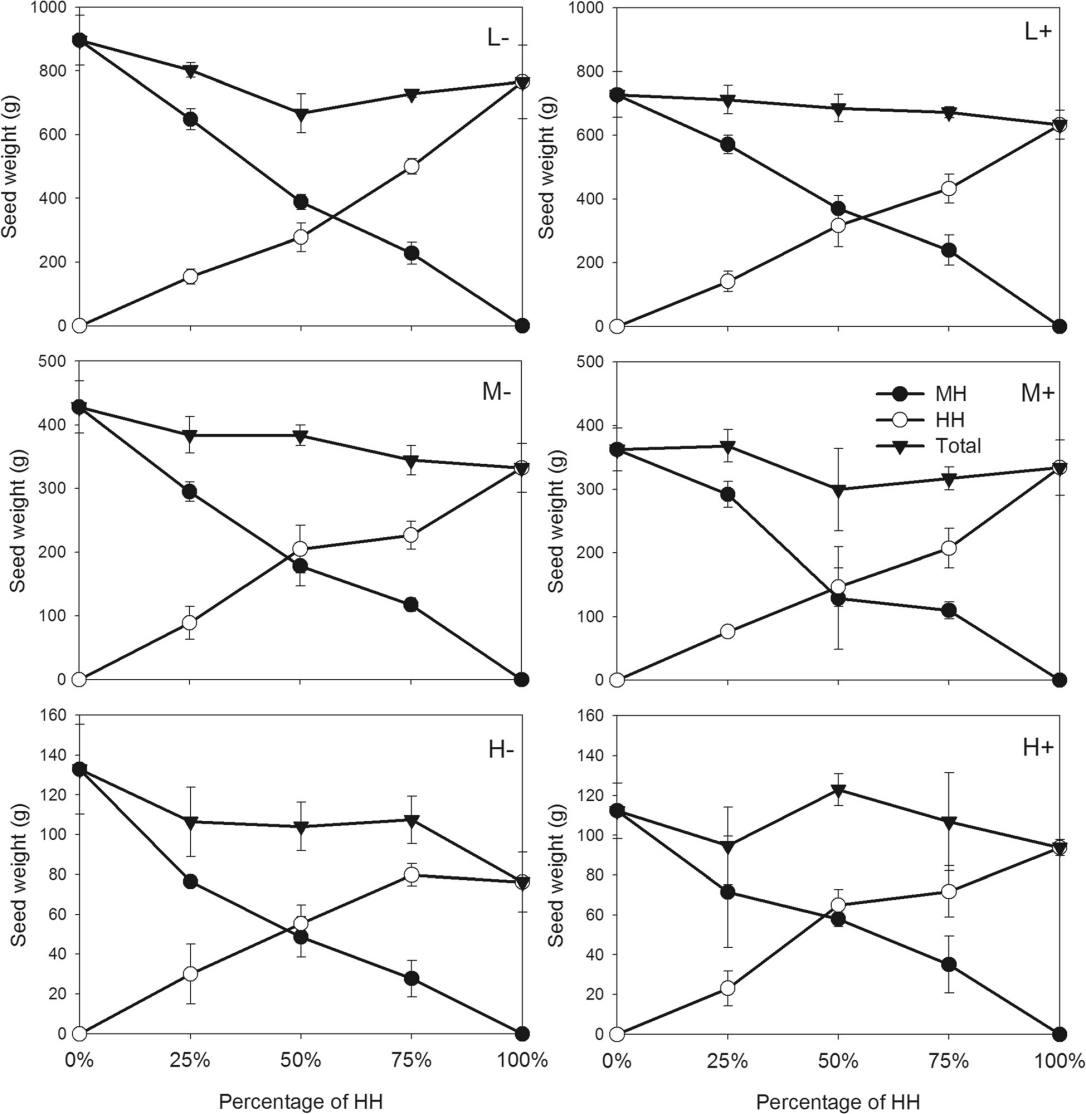
Total seed production per plot in terms of the percentage of HH in Wuhan site. Full circle symbols, total seed weight of all MH plants per plot; empty circle symbols, HH plants; full triangle symbols, all plants (including HH plus MH). L, M, and H indicate low, medium and high densities respectively. “+” and “-” indicate the presence and absence of infection. Vertical bars denote SEM.

**Figure 4:**
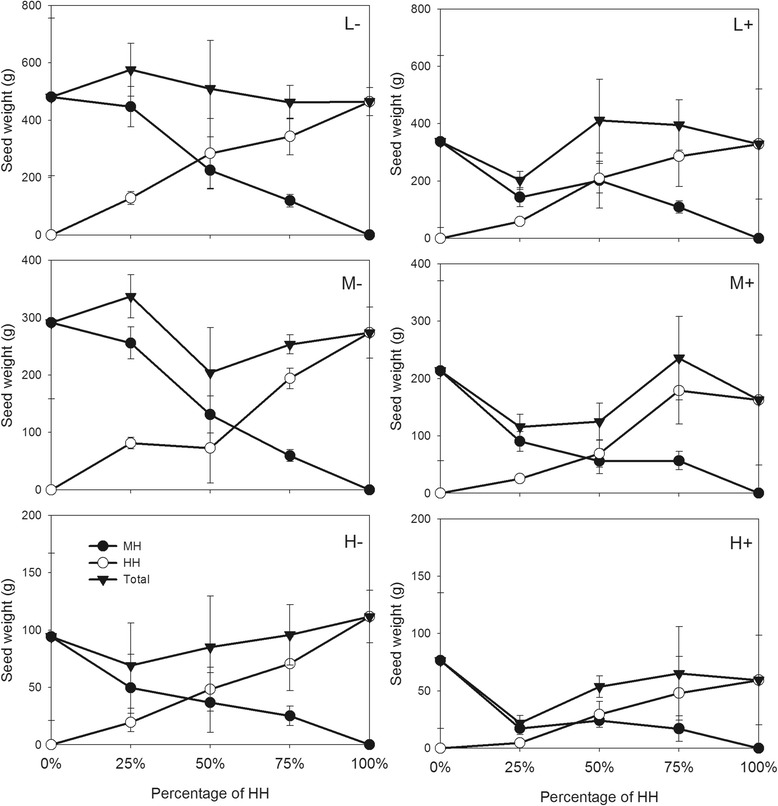
Total seed production per plot in terms of the percentage of HH in Nanchang site. Full circle symbols, total seed weight of all MH plants per plot; empty circle symbols, HH plants; full triangle symbols, all plants (including HH plus MH). L, M, and H indicate low, medium and high densities respectively. “+” and “-” indicate the presence and absence of infection. Vertical bars denote SEM.

Plot production for biomass and seed weight was lower in the Nanchang site than that in the Wuhan site. The variation of population yield among different percentages of HH was higher in the Nanchang site than that in the Wuhan site (Figures 3, 4), suggesting that the population in the natural habitat was more dynamic than that in the habitat under human control, for both transgenic and non-transgenic rice.

Overall plot productivity biomass did not vary among different percentages of transgenic rice in the plots. The plot-level analysis showed little difference among pure plots and mixtures of the HH and MH plants for total biomass in Wuhan and Nanchang and for seed weight in Nanchang (Table 4; Figures 3, 4;). Total seed weight per plot decreased with increasing percentage of HH in Wuhan site (Table 4, Figure 3). Overall performance per plot including all the HH and MH plants was higher in low density than in high density (Table 4). Infection significantly decreased total seed weight of all the HH and MH plants per plot in the two sites, but had little effect on total biomass (Table 4). The insect damage and plant competition between transgenic and non-transgenic rice showed an additive effect on population production. A significant interaction existed between infection and density for the total seed weight in the two sites and the biomass in Wuhan site. Little interaction, however, was found among infection, HH percentage and density, except for seed weight in Wuhan (Table 4).

**Table 4:** **Split-plot ANOVA for total biomass and seed weight per plot in Wuhan and Nanchang sites**

		**Wuhan**		**Nanchang**	
	**DF**	**Biomass**	**Seed weight**	**Biomass**	**Seed weight**
Infection (I)	1	5.70	249^**^	4.84	20.8^*^
Percentage (P)	4	0.43	6.60^**^	1.45	0.17
I × P	4	0.38	1.61	1. 25	1.15
Density (D)	2	1379^***^	1943^***^	729^***^	147^***^
I × D	2	3.64^*^	7.66^*^	1.11	5.08^*^
P × D	8	1.07	2.11	1.31	0.90
I× P × D	8	0.51	2.28^*^	1.31	0.86

^*^, ^**^and ^***^ indicate significant difference at the P < 0.05, P<0.01 and P<0.001 levels.

### Insect survival under the coexistence of transgenic and conventional rice

**Figure 5:**
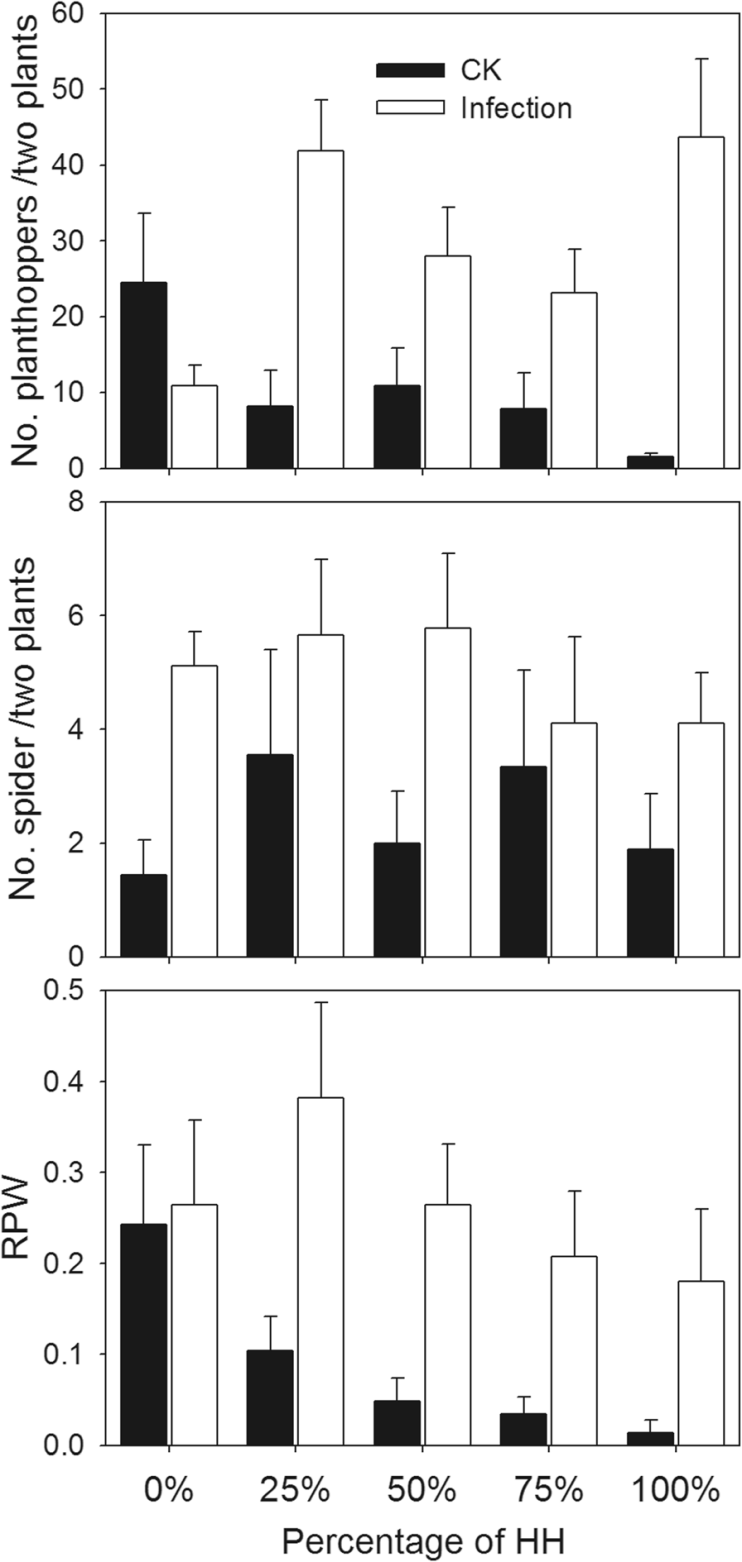
Insect number per two plants against the percentage of HH in Nanchang site. RPW, rate of plants having white straws, representing *C. suppressalis* damage. White bars, infection; black bars, control.

The coexistence of transgenic and conventional rice had little effect on non-target insects in the simulated natural environment. There were more insects in infected plots than in the plots without infection in the Nanchang site, i.e., 29.5 *vs.* 10.6 planthoppers per two plants on average and 5.0 *vs.* 2.4 spiders per two plants on average (Figure 5). The number of planthoppers and spiders and the rate of infected plants by *Chilo suppressalis* (RPW) did not differ significantly among different percentages of HH. The RPW decreased with the increased percentage of HH plants in the plots without infection.

## Discussion

### Effects of coexistence on plant growth and reproduction

The coexistence of transgenic and conventional rice has an effect on plant growth and reproduction, which depends on the local environment, herbivory festation and competition. In the agricultural ecosystem with insecticides to control non-targeted insects (Wuhan site), transgenic rice produced more aborted seeds and less viable seeds than non-transgenic plants. No difference of aborted and viable seeds was observed between transgenic and non-transgenic plants in the natural ecosystem, where non-targeted insects existed without insecticide application (Nanchang site). In another study, transgenic MH86 (a restorer line) with *Bt/CPTI* showed a yield loss in comparison with its non-*Bt* counterpart under low insect pressure [28,29], but little yield differences were observed between *cry1Ab/c Bt*-Shanyou63 (a hybrid variety) and its non-*Bt* counterpart [30,31]. Wang *et al*. [32] found *Bt*-transgenic Minghui63 with *cry2A* and *cry1C* had lower grain yield than Minghui63. They held that this yield loss was correlated with reduced grain filling percentage resulting from reduction of growth-promoting phytohormones. An alternative explanation is the fitness cost of expressing *Bt* transgene in the absence of insects, which has been shown in rice [29] and rice hybrids [20]. No fitness cost of expressing *Bt* transgene in the absence of selection pressure was detected in *Brassica* [18,33], in sunflower or in rice [21].

As shown in this study, herbivory has a negative effect on plant growth and reproduction [34,35]. Certain studies, however, show little effect of herbivory on plant performance because of compensatory growth [36,37]. Here, insect damage (*Chilo suppressalis*) decreased seed weight but had little effect on seed number. Vaughton and Ramsey also found that defoliation reduced seed mass but not seed number [38]. Insect infection increased the number of aborted seeds in the natural ecosystem, in accordance with previous results that foliar herbivory increased the rate of aborted seeds while floral herbivory decreased it [39].

Plant density, representing competition intensity, significantly reduced the production of both GM and non-GM rice. Competition and herbivory had an additive effect on plant performance, as shown in other reports [2,40]. The interaction could be antagonistic because the impacts of herbivory on neighbors could decrease competition from neighbors [41,42]. There might be no interaction if the disadvantage of the susceptible plants failed to correlate with the specific or combined pressure from competition and herbivory [43].

### Relative competitive ability

Theoretically, plants harboring the *Bt* gene are expected to display insect-resistance and less herbivore damage, leading to greater competitive ability than insect-susceptible plants [18,19,21,44]. In this study, however, both RD and RCC values showed that *Bt*-transgenic rice failed to display competition advantage compared with non-transgenic rice. This is in agreement with other reports. For example, Chen *et al.* (2006) found no competition advantage of *Bt*-transgenic rice to control lines because of yield losses in transgenic rice [28].

There are four possible explanations for our failure to observe competitive advantage of *Bt*-transgenic rice. The first is the fitness cost of expressing *Bt* transgene discussed above. The second is that more non-target insects were observed in infected cages than in control cages. *Bt*-transgenic rice resisted to target insects but not to non-target insects (e.g. planthoppers), which affected the growth of *Bt*-transgenic rice. Thirdly, the presence of transgenic plants could protect non-transgenic plants, a “halo effect” proposed by Alstad & Andow [45] and observed in other reports [23,24]. Hutchison *et al*. [24] found that non-*Bt* corn was protected by the adjacent *Bt* corn from being damaged, resulting in billions of dollars of economic benefits for the United States over 14 years. *Bt* cotton has been cultivated over 10 years in Northern China, significantly decreasing the number of target insects (cotton bollworm, *Helicoverpa armigera*) on *Bt* cotton, non-*Bt* cotton and other host crops [23,46]. Fourthly, the relative competitiveness of insect-resistant plants to conventional plants depends on access to resources and allocation strategy. At Nanchang site, insect infection led to an increase in resources allocation to herbivory defence (including non-target insects), resulting in low production. Resistant plants have better access to resources (including light, water, nutrient) than the susceptible counterpart because the former suffers less damage. However, there is a trade-off between resistance capacity and competitive ability for plants e.g. [40], since the allocation of resources to defence will decrease plant growth [47]. Kalinina *et al.* found that transgenic wheat resistant to powdery mildew was inferior to its non-transgenic counterpart in competition even with the presence of the pathogen, since transgenic wheat reduced yield and seed number under competition compared with its control [48].

### Population yield in the coexistence of transgenic and conventional rice

Since volunteer plants could persist in or outside of cultivation through pollen-mediated gene flow or seed movement of transgenic plants [10-12], it seems inevitable that transgenic and non-transgenic plants would coexist in the field and natural habitats [3,7,13-16]. One concern of the coexistence is the shift from a susceptible population to a resistant one irrespective of insects e.g. [18].

*Bt*-transgenic rice failed to show competition advantage compared with non-transgenic rice in the present study. This would hamper the increased frequency of *Bt*-transgenic rice and encourage coexistence of transgenic and non-transgenic plants in mixed populations. In the Wuhan site, total seed weight per plot decreased with increased percentage of transgenic rice, due to the fitness cost of expressing *Bt* gene as discussed above. In an experiment to simulate insect-susceptible plants by mechanical wounding, the increase of healthy plants in a population of damaged plants did not increase the population productivity [2].

However, high fluctuation of population production in the natural ecosystem (Nanchang site) suggests its vulnerability, which might lead to a shift from non-transgenic to transgenic population and vice versa. Previous studies found that transgenic plants were more likely to invade a harsh environment [44,49]. The commercial planting of transgenic crops has increased non-target insects in the fields [23,46]. Such insect infestation is ubiquitous in natural habitats, which might facilitate the spread of insect-resistant transgenes. In this study, planthoppers increased when target insects (*C. suppressalis*) occurred, explaining high fluctuation of population production. In addition, rice volunteers frequently appear in the wastelands, orchards or other habitats near fields because of seed movement or seedlings dispersal during the farming. Thus, it is likely that cultivated transgenic rice establishes feral populations as a type of weedy rice if the transgene could persist in the nature over time.

## Conclusions

Considering the results of plant growth and reproduction, relative competitive ability and population yield, it is likely that the *Bt*-transgenic and non-transgenic rice will coexist in agricultural ecosystems when commercial release of transgenic crops is permitted. A population in natural habitats is also possible to shift from non-transgenic to transgenic plants. The agricultural benefits and population productivity in diverse cultivation systems with coexisting GM and non-GM crops have been addressed [7,14]. Coexistence allows crop growers and consumers to produce or purchase conventional, organic or GM crops. In addition, as gene flow occurs naturally through pollen or seed flow, a transgenic crop may establish a feral population or volunteers that persist in natural ecosystems, such as oilseed rape, maize, flax and rice [4,10,11,15,25,44]. To mitigate the adventitious presence of GM plants caused by gene flow, strategies based on breeding, agronomic or molecular methods have been proposed e.g. [50]. The evaluation of the ecological consequences of *ex post* coexistence with GM plants, particularly in the natural ecosystem, is essential to construct sound regulations to reduce the adventitious presence of GM plants in agroecosystem and the invasion of transgenes in natural ecosystems.

## Methods

### Plant material

*Bt*-transgenic rice (*Oryza sativa*) Huahui-1 (HH) was granted a biosafety certificate in 2009. HH and its non-transgenic counterpart Minghui-63 (MH), donated by Prof. Y. Lin from Huazhong Agricultural University, were employed in this study. *Bt*-transgenic rice Huahui-1 conferred a fusion gene *Cry1Ab/Cry1Ac* driven by actin-I promoter [30]. The expression of *Bt* toxin protein in 120 HH seeds was confirmed with PCR and enzyme-linked immunosorbent assay (ELISA) before sowing.

### Field trial

**Figure 6:**
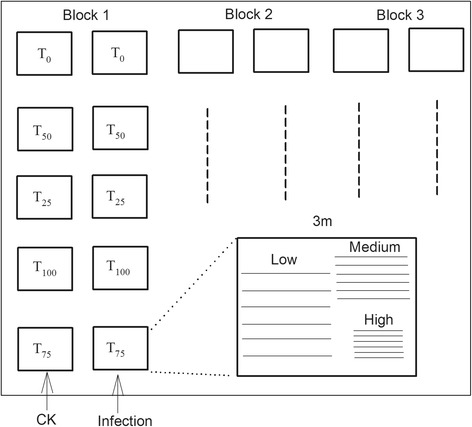
The map of the infection experiments in Wuhan and Nanchang sites. Infection, insect release of *C. suppressalis*; CK, without insect release. Low, medium and high densities with 0.3, 0.2 and 0.1 m space between plants. T_0_, T_25_, T_50_, T_75_ and T_100_ indicate the percentages of transgenic rice.

*Bt*-transgenic rice Huahui-1 (HH) and its counterpart Minghui-63 (MH) were sown in the fields. Rice seedlings were then transplanted into cages (3 m width × 3 m length × 2.5 m height, protected by a 2 mm-mesh nylon net), each cage containing three plots (6 × 6 = 36 plants per plot) with three densities: 0.30 m, 0.20 m and 0.10 m row spacing (Figure 6). There were thirty cages in total in five rows and six columns, and three-meter space was set between the cages to ensure the plants’ access to sunlight. To simulate the colonization process of insect-resistant plants into a non-transgenic population, five percentages of HH were added to plots in a replacement series: 0 (T_0_), 25% (T_25_), 50% (T_50_), 75% (T_75_), and 100% of HH (T_100_). The five percentages were placed randomly in a column (Figure 6). Plant types (HH or MH) were placed randomly in each plot, ensuring the same ratio of MH to HH for the 16 plants in the center and the 20 plants at the border. Thirty cages were separated into three blocks, with one column of cages infected by insects per block (Figure 6). Two second-instar larvae of *Chilo suppressalis* [Walker] were introduced to each plant.

The experiments were carried out at two sites, a transgenic rice research base of Huazhong Agricultural University, Wuhan, Hubei province (N30°27′58.8′′, E114°21′45.8′′), and a transgenic rice research base of Jiangxi Academy of Agricultural Sciences, Nanchang, Jiangxi province (N28°21′91.4′′, E115°55′49.7′′). The annual mean temperature and precipitation were 18.0°C and 2059 mm in Nanchang and 15.5°C and 1416 mm in Wuhan city in 2012 (China Statistical Yearbook). Rice seeds were sown in the fields on June 12, 2012 at Wuhan site and on June 5, 2012 at Nanchang site. The seedlings were transplanted in the fields after 28–30 days. After the seedlings survived, second-instar larvae of *C. suppressalis* were introduced to the plants. The site at Wuhan was considered as an agricultural ecosystem, with weeds removal by hands and insecticide spraying. Insecticide (22% a.i. buprofezin in 1000-fold dilution, 450 g ha^−1^) was applied twice (at the third and sixth week after infection) to control rice planthoppers in infected cages during the growing season. Simultaneously, avermectin (1.8 g L^−1^ a.i. in 1000-fold dilution, 2.5 L ha^−1^) and triazophos (20% a.i. in 1000-fold dilution, 1500 g ha^−1^) for *C. suppressalis* were applied twice in the cages without infection. The site at Nanchang simulated a natural ecosystem, without insecticide spraying or weed removal in the cages during the growing season. Fertilization and irrigation was implemented whenever need according to local agricultural farming.

### Measurements

Plant height, number of valid tillers, the rate of invalid tillers (dead tillers plus tillers without ears) to all tillers per plant (RIT), biomass (straw weight after being insolated ten days) and seed weight per plant were measured for all 16 plants (HH and/or MH) in the center of each plot. Reproductive allocation (RA) per plant was defined by dividing seed weight with the biomass. Six ears were chosen randomly for each plant type (HH *vs.* MH) in the plot center to measure ear length, number of viable seeds and hollow seeds per ear, and mean values were regarded as variables per plant.

Field insects (rice planthopper and spider) on two randomly selected plants per plot and the rate of plants with white straws (RPW, representing *C. suppressalis* damage) to 16 center plants (HH and/or MH) were surveyed before harvesting at the Nanchang site. In Wuhan, insects were not surveyed due to the application of insecticides.

### Statistical analysis

The mean values of all HH or MH plants per plot were used for statistical analysis. The data were *log*-transformed to ensure a normal distribution of residuals. Split-plot ANOVA (Y ~ Site + I × P × D × T+ Error (block/I/P/D)) was employed to test the effects of site (Wuhan *vs.* Nanchang), insect infection (I), percentage of HH plants (P), plant density (D) and plant types (T, HH vs. MH). Due to significant site effects, split-plot ANOVA (Y ~ I × P × D × T+ Error (block/I/P/D)) was used in Wuhan and Nanchang, respectively. Tukey’s HSD test was used for multiple comparisons among percentages of HH plants.

To further investigate the differences between HH and MH individual plant biomass, relative to the MH performance in pure stand T_0_, the relative differences RD = (R_Ti_ - S_Ti_)/S_T0_ were calculated (value at T_100_ was calculated as RD = (R_T100_ - S_T0_)/S_T0_) [2]. RD values were subject to a *t*-test to determine their deviation from 0.

For replacement series analysis, the relative crowding coefficient (RCC) was calculated according to the equation in Ramachandran *et al.* [18]:

$$ \mathrm{R}\mathrm{C}\mathrm{C} = \left(\sum \left({\mathrm{R}}_{\mathrm{T}\mathrm{i}}/{\mathrm{S}}_{\mathrm{T}\mathrm{i}}\right)/3\right)/\left({\mathrm{R}}_{\mathrm{T}100}\right./\left.{\mathrm{S}}_{\mathrm{T}0}\right) $$

where R_Ti_ and S_Ti_ are the biomass values for HH and MH respectively at percentage T_i_ (T_25_, T_50_ and T_75_) per unit area in the plot center. An RCC value of 1 indicates equal competitiveness between R and S plants. RCC >1 indicates that R plants are more competitive than S plants and vise versa. A *t*-test was performed to determine whether the RCC values were significantly deviated from 1.

As for biomass and full seed weight, a per-plot ANOVA (Y ~ I × D × P+ Error (block/I/D)), with block as a random factor and infection, density and percentage of HH plants as fixed factors, was carried out using the sum of the data of all MH and HH plants per plot, i.e. the whole population production. All statistic analyses were conducted in R software [51].
